# Ulcerated gastrointestinal stromal tumor causing a gastrogastric intussusception

**DOI:** 10.1002/ccr3.2662

**Published:** 2020-01-22

**Authors:** Jan Vandereycken, Naomi Michotte, Frederik Vandenbroucke, Johan de Mey

**Affiliations:** ^1^ Radiology Department UZ Brussel Jette Belgium; ^2^ Internal Medicine Department UZ Brussel Jette Belgium

**Keywords:** gastrogastric, GIST, imaging, intussusception, invagination

## Abstract

When a gastrointestinal intussusception is found, an underlying lesion should be excluded.

## Graphical abstract

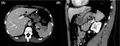

A 46‐year‐old woman was referred to the emergency department with severe epigastric pain. Physical examination, electrocardiogram, chest X‐ray, and laboratory findings were normal. A contrast‐enhanced computed tomography (CT) (Figure 1) was performed. The scan showed an invagination of the stomach wall. The diagnosis of an organo‐axial gastrogastric intussusception was made, probably caused by a well‐described round mass of 3 cm. This lesion showed a deep ulceration with an air‐fluid level (Figure 1).

**Figure 1:**
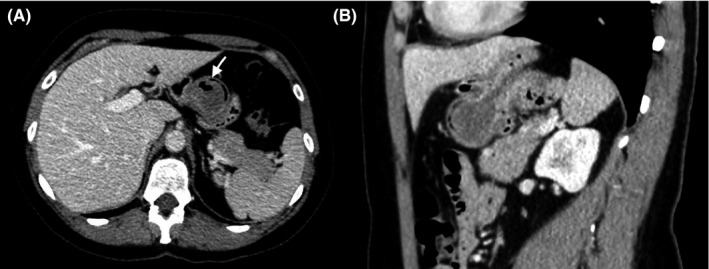
Contrast‐enhanced computed tomography of the upper abdomen. Axial image (A) showing an ulcerated mass originating from the stomach with a small air‐fluid level (arrow). Sagittal image (B) showing an organo‐axial gastrogastric intussusception

The patient underwent gastric endoscopy and biopsy. An ulcerated submucosal mass at the gastric fundus (Figure 2) was seen. Echo‐endoscopy (Figure 2) showed a single sharply delineated submucosal hypo‐echoic mass. The biopsy confirmed the diagnosis of a benign gastrointestinal stromal tumor (GIST). A laparoscopic partial gastrectomy was performed.

**Figure 2:**
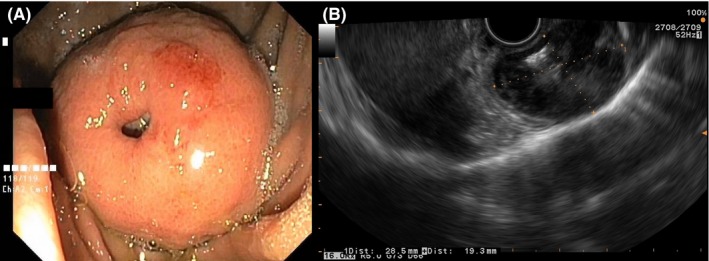
Gastric endoscopy (A) showing a well‐rounded mass with central ulceration. Echo‐endoscopy (B) showing a hypo‐echoic content of this submucosal mass

Intussusception or invagination of the proximal gastrointestinal tract is uncommon, and only few gastrogastric intussusceptions have been reported. Most intussusceptions have been reported in children, where 90% of cases are idiopathic. In adults however, in up to 90% of cases an underlying pathology could be found. Neoplasms account for 65% of all causative lesions in adults.^1^ Gastrointestinal stromal tumors (GIST) have been found anywhere along the gastrointestinal tract but are more frequent in the stomach, accounting for 60% of cases.^2^

## CONFLICT OF INTEREST

None declared.

## AUTHOR CONTRIBUTIONS

JV: made diagnosis, obtained and edited images, and prepared and drafted the manuscript. NM: obtained images and reviewed the manuscript. FV and JDM: reviewed the manuscript.

## References

[1] Choi SH , Han JK , Kim SH , et al. Intussusception in adults: from stomach to rectum. Am J Roentgenol. 2004;183:691‐698. 15333357 10.2214/ajr.183.3.1830691

[2] Akahoshi K , Oya M . Gastrointestinal stromal tumor of the stomach: How to manage? World J Gastrointest Endosc. 2010;2(8):271‐277. 21160626 10.4253/wjge.v2.i8.271 PMC2998840

